# Psychopathology and Suicide among Quebec Physicians: A Nested Case Control Study

**DOI:** 10.1155/2011/936327

**Published:** 2011-07-28

**Authors:** Pierre Gagné, Javad Moamai, Dominique Bourget

**Affiliations:** ^1^Clinique Médico-Légale de l'Université Sherbrooke, 234 Dufferin, Bureau 300, Sherbrooke, QC, Canada J1H 4M2; ^2^Centre Hospitalier Pierre-Janet, 20 Pharand, Université d'Ottawa, Gatineau, QC, Canada J9A 1K7; ^3^Royal Ottawa Mental Health Centre, University of Ottawa, 1145 Carling Avenue, Ottawa, ON, Canada K1Z 7K4

## Abstract

*Objective*. To describe a psychiatric profile and characteristics of physicians who killed themselves in Quebec between 1992 and 2009. *Method*. The cases of 36 physicians (7 females and 29 males) and 36 nonphysicians who committed suicide were matched for age and gender and examined in a nested case control design. All subjects were judged as definite suicide by the Quebec Coroner Head Office. Consensus regarding DSM-IV diagnoses was established by two forensic psychiatrists. *Results*. Rates of all Axis I diagnoses were 83% for physicians and 91% for nonphysicians at the time of suicide. Major depressive disorders were the most frequently observed pathology in both groups (61% and 56%, resp.). *Conclusions*. Physicians and nonphysicians who committed suicide in Quebec suffered from the same type of psychiatric disorder at the time of killing themselves. The findings advocate strongly for more efficient suicide prevention measures including early detection and treatment of mood disorders for the physicians.

## 1. Introduction

 Suicide, a complex public health problem, is the ninth leading cause of death in Canada [1]. WHO office on preventive practices in suicide and attempted suicide in its 1986 report, quoted by Nordentoft [2], defined suicide as “Suicide is an act with a fatal outcome which the deceased knowing or expecting a fatal outcome had initiated and carried out with the propose of provoking the change that he desired.” Suicidal acts can be considered as severe and preventable complications to a range of diseases and social conditions [2]. It is reported that 85%–90% of people who kill themselves have been suffering from some type of psychiatric disorders [3].

Previous research on physician suicide has been marked by inconsistent findings and has generated debate in several countries [4–16]. Although several studies [5, 7, 9, 11, 13] found a moderately higher risk of suicide among male physicians and a significantly higher risk of suicide among female physicians than in the general population, others have reported different results. Arnetz et al. [16] determined that only Swedish female physicians were more prone to suicide than the general population. In a Finnish study, Lindeman et al. [14] reported standardized suicide mortality ratios of 0.9 for male physicians and 2.4 for female physicians. Hawton et al. [11] found a significantly lower standardized suicide mortality ratio of 0.67 for male physicians and a ratio of 2.02 for female physicians in the UK. More recent research [10] suggests that the disparity between physician and nonphysician suicide rates may be smaller. It has become evident that age and gender are major confounding factors in this area, which could partly explain the varied conclusions in the literature. For example, a recent study [4] reported that suicide rates were elevated for white female physicians and older white male physicians. These authors found a strong trend for increasing suicide ratio for male physicians with increasing age. 

 Some studies have attempted to determine why the medical profession is at high risk for suicide and why some physicians kill themselves and others do not. Physician suicide has been linked to financial difficulty [17], relationship [17, 18] and occupational [19] problems, marital status [12, 17], drug abuse and alcoholism [20–22], and preexisting psychiatric illness [18, 19, 21, 23].

 Although suicide is a multidimensional concomitant of psychiatric illness, the association between physician suicide and psychopathology has not been investigated extensively. Two psychological studies [18, 20] that addressed this issue found a relation between depression and suicide among physicians. However, neither study employed a control group of nonphysicians, limiting the generalizability of their findings. A difficulty inherent to an examination of suicide is that the subject cannot be evaluated and risk factors cannot be established. Although it may be challenging to identify appropriate control subjects in this regard, comparing physician and nonphysician suicide may reveal differences in existing psychopathology. Hawton et al. [24] emphasized the value of the psychological autopsy method in furthering an understanding of factors contributing to suicide and noted that use of a control group would aid in identifying specific risk factors for suicide. 

 Nearly ten Canadians kill themselves every day. Canada's suicide rate (14 per 100,000 population) is thirteenth in the 22 western industrialized countries. With a rate of 21 per 100,000 population, Quebec has the highest age-standardized provincial suicide rate in Canada [25].

 Several studies have noted a strong association between mental disorders and suicide. A systematic review [26] found that the median percentage of suicide victims with a mental disorder was 91 percent. Fifty-nine percent of those victims had an affective disorder. A landmark study [27, 28] on the relation between suicide and mental disorders in young men in Quebec reported that subjects who committed suicide suffered more clinical DSM-III-R disorders than living controls (88% versus 37%, resp.). These disorders included major depression, alcohol and drug dependence, and borderline personality disorder.

 Specific epidemiological studies are needed to obtain adequate evidence for the presence of psychopathology among physicians who committed suicide in the province of Quebec. In any given occupation, the risk of suicide being a low-frequency event associated with age and gender changes its pattern over time [29]. We therefore believe a case control study using the psychological autopsy method and including both physicians and general population subjects who committed suicide controlling for these three confounding factors, could be meaningful.

 The main objective of this study was to answer the following two questions: (1) are there any differences in psychopathology, measured by the presence of concurrent psychiatric disorders, between physicians' suicides and suicides by nonphysicians? and (2) are there any differences in treatment seeking rates for concomitant psychiatric illness between these two groups? We also attempted to explore possible differences in specific associated characteristics, such as methods used to commit suicide and circumstances pertaining to the situation, as secondary variables.

## 2. Method

 As suggested by Maris [30], we defined suicide when: “first, there was a death; second, the death was achieved by the individual who died; third, the death was intentional; and fourth, there was an active agent”. Quebec law requires that all possible suicide cases in the province be reviewed by the Coroner's Office. Access to the relevant material was granted authorization by the Quebec Coroner Head Office. The unique role of the coroner in Quebec allows public access to the coroner's investigative report and dispensation from the application of the general rules of confidentiality pertaining to personal data in order to facilitate study, teaching, or scientific purposes. The study was conducted in accordance with the guidelines of the Declaration of Helsinki. 

 A nested case control design was used to investigate the cohort of all suicide cases judged as definite suicide by the Quebec Chief Coroner Office. Between 1992 and 2009, there were on average 1300 suicides per year in Quebec. In each case, the coroners' files typically contained information on the individual's characteristics, the circumstances of death, the spatial location of the suicide, and the type of method used in the suicide; the coroner's report for the particular death, including opinion and recommendations; the police investigative report; the autopsy report; biochemical laboratory findings. Medical and psychiatric records were also part of the files when available. 

 We identified 36 cases of physician suicide and selected 36 controls (cases of nonphysician suicide) from the cohort, matching for age (±1 year), gender, and year of death (±4 years). This resulted in a sample size of 72 and a ratio of 1 : 1 physicians to nonphysicians.

 All legal records from coroners' definite suicide files were reviewed and compiled by the same two investigators (PG and DB), coroners with a medical specialty in psychiatry. These investigators established diagnoses by consensus, using available data, based on DSM-IV classification [31]. Review and analysis of the data were performed in an anonymous manner.

 Statistical analysis was conducted using SPSS for Windows (version 15.0) [32]. Comparisons of the physician group and the nonphysician group were made using odds ratios (OR) to estimate differences on psychiatric diagnosis and psychiatric service contact, with 95% confidence intervals (95% CI). Due to a relatively small number of suicide physicians and missing data, we did not make any statistical analysis for associated variables. These results were reported only descriptively as indication of tendency.

No information pertaining to the two-principal study variables was missing. However, data were missing for 10 of the 16 selected variables, with an average of 15% (6.7%–16.7%). The rates of missing data were nondifferential among the physicians and nonphysicians.

## 3. Results

### 3.1. Baseline Characteristics of Physicians

Table 1 shows characteristics of the 7 female and 29 male physicians in our sample. These physicians represent on average 2.6% of all definite suicides committed in Quebec during the study period. Median age at death was 50 years. Female physicians were significantly younger than male physicians at the time of suicide (*F* = 7.54, df = 1, *P* = 0.01). The physicians had practiced medicine for an average of 23 years. Almost two thirds of the physicians were practicing family medicine, and the others were practicing various specialties when they committed suicide. Most of the physicians (77.8%) were living in urban areas.

### 3.2. Comparison between Physician and Nonphysician Suicide

Table 2 shows the similarities between the physicians and nonphysicians in the pattern of concomitant psychiatric diagnoses based on DSM-IV classification. Psychiatric service contact is defined as any contact with a psychiatrist prior to the completed suicide. In contrast to any lifetime contact with a psychiatrist, this is meant to say that the subject specifically consulted (either receiving a medication or not) a psychiatrist in relation to his/her current episode of illness.

 Most of the physicians (83.3%) were suffering from an identifiable psychiatric disorder at the time of their suicide. Major depressive disorders (61.1%) were the most common diagnoses followed by bipolar disorders (8.3%) and schizophrenia (2.8%). Two thirds (66.7%) of the physicians sought psychiatric help before their suicide attempt. No significant difference in psychopathology and help-seeking was found between the two groups.

### 3.3. Demographic Characteristic

 Most of the physicians (97.2% versus 100%) were Caucasian. One male physician was African-American. Six of the physicians (16.7%) were married, 10 (27.8%) were living with a common-law partner, seven (19.4%) were divorced, and seven (23.3%) were single. A higher tendency of physicians to live with a partner (51.6% versus 31.4%) was observed. 

### 3.4. Circumstances of Suicide

 Fewer physicians than nonphysicians had issued a suicide warning (19.4% versus 44.4%). Almost one third of the physicians (27.8% versus 38.9%) made at least one prior suicide attempt. Thirty three percent of physicians (versus 38.9% for nonphysicians) left behind an explicative note. Physicians killed themselves mainly in their own home (77.8% versus 91.7%).

 Hanging was the most common method of suicide in both groups. Three physicians (8.3%) killed themselves by insulin injection. There was no difference for specific method of suicide. However, the absolute percentage difference between use of firearm and suicide was less marked for the physicians than the nonphysicians (5.6% versus 13.9%).

### 3.5. Pattern of Recent Problems

 Physicians who committed suicide had less identified difficulty in their relationships with others and with their environment than had nonphysicians (38.9% versus 75.5%). They had less frequent financial (16.7% versus 31.6%) and almost same rate of legal problems (16.7% versus 13.9%) as had nonphysicians. Of these six physicians, two were under investigation for malpractice. Physicians had a lesser tendency for physical health problems (27.8% versus 36.1%) and had alcohol and drug problems (19.4% versus 33.3%) than the nonphysicians.  Table 3 illustrates relevant demographic, psychosocial and clinical findings, as described above, in the two comparison groups.

## 4. Discussion

 This is the first Canadian study to examine psychopathology and associated characteristics of suicide among Quebec physicians. Our results are highly applicable to other physician populations living outside Quebec, especially in the western societies having same religious, cultural characteristics, and same health care system. It should be noted that there is no private psychiatric care in Quebec.

 The physicians in this sample were similar in many ways to nonphysicians who killed themselves in Quebec. This is in accordance with studies from other countries [18, 20]. Our research confirms the important association between psychopathology and suicide. Our study is unique in that the data demonstrate that physicians who committed suicide were suffering from psychiatric disorders as frequently as nonphysicians who killed themselves. Both groups were mainly experiencing major mood disorders (physicians 69.4%; nonphysicians 61.2%); more specifically, major depressive disorders (61.1% and 55.6%, resp.). 

 Major depressive disorder is a highly prevalent, severe, and disabling disorder. A substantial gap in the treatment of this disorder is a serious concern [33]. Depression is likely an important factor contributing to physician suicide and must be considered to enable the development of strategies to reduce suicide rates among physicians [23, 34, 35]. The mental health of physicians continues to be a topic of considerable concern [36–38]. As stated by Myers and Gabbard [3], “Suicide prevention is central and pivotal in decreasing the numbers of doctors who kill themselves each year.” A study conducted in Ontario [39] reported that depression was three to four times more prevalent among family medicine residents than in the general population. Despite this fact, nearly one third of physicians in our study did not have any prior contact with psychiatric services. This finding may be explained by the likelihood that the stigma attached to mental illness is greater within the field of medicine than in the general population [36]. Moreover, physicians who commit suicide may endorse the myth that psychiatric treatment has nothing to offer them [40]. Physicians do not adequately diagnose depression in themselves and their colleagues, and they do not seek treatment as they do for other health conditions. Physicians may try to hide their condition to protect their careers [41]. 

 As suggested by the consensus statement of the American Medical Association [23], professional attitudes and institutional policies need to be changed to encourage physicians with mental health problems to seek help. They should be informed of their rights, privileges, and obligations regarding disclosure of a psychiatric diagnosis and treatment [41]. We should continue to increase physicians' awareness of warning signs of suicidal ideation, such as observable signs of serious depression (which may be found in references [20, 33]). As Preven [42] noted long ago, “If the physician can not heal himself, perhaps he can learn to recognize the need for assistance.” (page 61).

 We found that fewer physicians than nonphysicians issued a warning for their acts. Physicians also had less previous suicide attempts than nonphysicians, although the difference did not reach statistical significance. This finding raises the question of impulsivity [43] and “not a cry for help” [34] in suicide prevention [43]. It has been suggested [44] that impulsivity may play a far greater role in suicide than previously thought. According to Bridge [44], 75 percent of suicidal subjects took less than one hour to make the decision to end their own life.

 Similar to findings of other studies [20, 45], more than one third of physicians committed suicide by self-poisoning. The availability of drugs and knowledge of their effect is a professional risk factor unique to this population [5, 20, 46]. In our study, 19 percent of physicians had drug and alcohol problems, a rate similar to that of all physicians in North America [22]. 

 The Quebec female physicians killed themselves in much younger age than their male colleagues (41 versus 51 years).

 This study has some methodological limitations. Although the psychological autopsy method is known to be useful [24] in determining factors potentially contributing to suicide, the validity of this method is controversial. Furthermore, information contained in official death entries was limited. For instance, data were minimal regarding severity of physical health conditions, relationship difficulty, financial problems, and drug abuse. The most serious methodological problem was the amount of missing information on several selected variables. The analyses were based on definite cases and not on probable or unknown cases. There is, therefore, the possibility of underestimation of the magnitude of a relationship, as well as a possible risk of Type II error. However, it is unlikely that this problem would change the overall direction of conclusions. Because we examined the entire Quebec suicide population occurring over a 17-year period, taking care that clinical reviewers collected and analyzed data in an anonymous manner, the possibility of selection bias is slim.

 In spite of a relatively small number of suicide physicians, the sample size is the largest possible since it is based on the entire Quebec physician suicide population and a gender age and year of death matched control group from a fairly homogenous society. An 1 : 1 case-control design was therefore opted to prevent Type I error. Our results also follow trends indicated by previous research. Therefore, we feel confident that our results are reliable. While acknowledging the methodological limitations of our study, our findings indicate that physicians and nonphysicians who commit suicide in Quebec suffer from the same type of psychiatric disorder at the time that they kill themselves. This main finding and our observations that female doctors killed themselves in younger age, taken together with the fact that number of women entering medicine is increasing, advocate strongly for more efficient suicide prevention measures for the physicians.

##  Conflict of Interests

The authors report no conflict of interests.

## Figures and Tables

**Table 1:** Baseline characteristics of physicians.

Characteristics	Women	Men	All
	*n* = 7	*n* = 29	*n* = 36
Age at death*	41 ± 6	51 ± 5.2	50 ± 6.8
Range	26–53	33–67	26–67
Living in urban areas	42.9%	86.2%	77.8%
Duration of active practice*	18 ± 7.5	24 ± 6	23 ± 5.8
Range	1–19	9–40	1–40
Medical specialty (*n*):			
Family physician	5	20	25
Radiology	—	3	3
Psychiatry	—	2	2
Other**	2	4	6

*Median of year ± semi-interquartile range.

**Cardiology, dermatology, nephrology, pathology, pediatrics, and rheumatology.

**Table 2:** Concomitant psychiatric diagnoses.

Psychiatric diagnoses	Physicians	Controls	OR	95% CI
	*n* = 36	*n* = 36		
DSM-IV code:				
Any	83.3%	91.7%	0.46	0.10–1.98*
Major depressive disorders	61.1%	55.6%		
Bipolar disorders	8.3%	5.6%		
Schizophrenia	2.8%	8.3%		
Other	11.1%	22.2%		
Psychiatric service contact	66.7%	72.2%	0.79	0.28–2.01*

*No significant difference.

**Table 3:** Selected associated characteristics.

Variable	Physicians	Controls
	*n* = 36	*n* = 36
Age, (years, median)	50	49.5
Gender, male	80.6%	80.6%
Ethnicity, white	97.2%	100.0%
Live with a partner	51.6%	31.4%
Pattern of recent problems:		
Relationship	38.9%	75.0%
Physical health	27.8%	36.1%
Alcohol and drugs	19.4%	33.3%
Legal	16.7%	13.9%
Financial	16.7%	31.6%
Suicide warning	19.4%	44.4%
Suicide note	33.3%	38.9%
Past suicide attempts	27.8%	38.9%
Location of suicide:		
Hospital	5.6%	2.8%
Home	77.8%	91.7%
Hotel	11.1%	0.0%
Other	5.6%	5.6%
Method of suicide:		
Hanging	41.7%	50.0%
Poisoning (drugs)	30.6%	16.7%
Gas (carbon monoxide)	8.3%	8.3%
Firearm	5.6%	13.9%
Other	13.9%	11.1%

## References

[1] http://www40.statcan.gc.ca/l01/cst01/health30a-eng.htm?sdi=mortality%20rates.

[2] Nordentoft M (2007). Prevention of suicide and attempted suicide in Denmark: epidemiological studies of suicide and intervention studies in selected risk groups. *Danish Medical Bulletin*.

[3] Myers MF, Gabbard GO (2008). The suicidal physician and aftermath of physician suicide. *The Physician as Patient*.

[4] Petersen MR, Burnett CA (2008). The suicide mortality of working physicians and dentists. *Occupational Medicine*.

[5] Torre DM, Wang NY, Meoni LA, Young JH, Klag MJ, Ford DE (2005). Suicide compared to other causes of mortality in physicians. *Suicide and Life-Threatening Behavior*.

[6] Schernhammer E (2005). Taking their own lives—the high rate of physician suicide. *New England Journal of Medicine*.

[7] Schernhammer ES, Colditz GA (2004). Suicide rates among physicians: a quantitative and gender assessment (meta-analysis). *American Journal of Psychiatry*.

[8] Hem E, Haldorsen T, Aasland OG, Tyssen R, Vaglum P, Ekeberg Ø (2005). Suicide rates according to education with a particular focus on physicians in Norway 1960-2000. *Psychological Medicine*.

[9] Stack S (2004). Suicide risk among physicians: a multivariate analysis. *Archives of Suicide Research*.

[10] Innos K, Rahu K, Baburin A, Rahu M (2002). Cancer incidence and cause-specific mortality in male and female physicians: a cohort study in Estonia. *Scandinavian Journal of Public Health*.

[11] Hawton K, Clements A, Sakarovitch C, Simkin S, Deeks JJ (2001). Suicide in doctors: a study of risk according to gender, seniority and specialty in medical practitioners in England and Wales, 1979–1995. *Journal of Epidemiology and Community Health*.

[12] Aasland OG, Ekeberg Ø, Schweder T (2001). Suicide rates from 1960 to 1989 in Norwegian physicians compared with other educational groups. *Social Science and Medicine*.

[13] Frank E, Biola H, Burnett CA (2000). Mortality rates and causes among U.S. physicians. *American Journal of Preventive Medicine*.

[14] Lindeman S, Läärä E, Hirvonen J, Lönnqvist J (1997). Suicide mortality among medical doctors in Finland: are females more prone to suicide than their male colleagues?. *Psychological Medicine*.

[15] Lindeman S, Läärä E, Hakko H, Lönnqvist J (1996). A systematic review on gender-specific suicide mortality in medical doctors. *British Journal of Psychiatry*.

[16] Arnetz BB, Horte LG, Hedberg A (1987). Suicide patterns among physicians related to other academics as well as to the general population. Results from a national long-term prospective study and a retrospective study. *Acta Psychiatrica Scandinavica*.

[17] Stack S (2000). Suicide: a 15-year review of the sociological literature—part I: cultural and economic factors. *Suicide and Life-Threatening Behavior*.

[18] Hawton K, Malmberg A, Simkin S (2004). Suicide in doctors: a psychological autopsy study. *Journal of Psychosomatic Research*.

[19] Agerbo E, Gunnell D, Bonde JP, Bo Mortensen P, Nordentoft M (2007). Suicide and occupation: the impact of socio-economic, demographic and psychiatric differences. *Psychological Medicine*.

[20] Lindeman S, Heinänen H, Väisänen E, Lönnqvist J (1998). Suicide among medical doctors: psychological autopsy data on seven cases. *Archives of Suicide Research*.

[21] Tyssen R, Vaglum P (2002). Mental health problems among young doctors: an updated review of prospective studies. *Harvard Review of Psychiatry*.

[22] Gold MS, Frost-Pineda K, Melker RJ, Schernhammer E (2005). Physician suicide and drug abuse. *American Journal of Psychiatry*.

[23] Center C, Davis M, Detre T (2003). Confronting depression and suicide in physicians: a consensus statement. *Journal of the American Medical Association*.

[24] Hawton K, Appleby L, Platt S (1998). The psychological autopsy approach to studying suicide: a review of methodological issues. *Journal of Affective Disorders*.

[25] Langlois S, Morrison P (2002). Suicide deaths and suicide attempts. *Health Reports*.

[26] Cavanagh JTO, Carson AJ, Sharpe M, Lawrie SM (2003). Psychological autopsy studies of suicide: a systematic review. *Psychological Medicine*.

[27] Grunberg F, Lesage AD, Boyer R (1994). Suicide among young male adults in Quebec: psychopathology and utilization of medical services. *Sante Mentale au Quebec*.

[28] Lesage AD, Boyer R, Grunberg F (1994). Suicide and mental disorders: a case-control study of young men. *American Journal of Psychiatry*.

[29] Stack S (2001). Occupation and suicide. *Social Science Quarterly*.

[30] Maris RW (2002). Suicide. *Lancet*.

[31] American Psychiatric Association (1994). *Diagnostic and Statistical Manual of Mental Disorders*.

[33] Kessler RC, Berglund P, Demler O (2003). The epidemiology of major depressive disorder: results from the National Comorbidity Survey Replication (NCS-R). *Journal of the American Medical Association*.

[34] Hem E, Grønvold NT, Aasland OG, Ekeberg Ø (2000). The prevalence of suicidal ideation and suicidal attempts among Norwegian physicians. Results from a cross-sectional survey of a nationwide sample. *European Psychiatry*.

[35] Frank E, Dingle AD (1999). Self-reported depression and suicide attempts among U.S. women physicians. *American Journal of Psychiatry*.

[36] Worley LLM (2008). Our fallen peers: a mandate for change. *Academic Psychiatry*.

[37] Pitt E, Rosenthal MM, Gay TL, Lewton E (2004). Mental health services for residents: more important than ever. *Academic Medicine*.

[38] Field R, Haslam D (2008). Do you have your own doctor, doctor? Tackling barriers to health care. *British Journal of General Practice*.

[39] Earle L, Kelly L (2005). Coping strategies, depression, and anxiety among Ontario family medicine residents. *Canadian Family Physician*.

[40] Shortt SED (1979). Psychiatric illness in physicians. *Canadian Medical Association Journal*.

[41] Hampton T (2005). Experts address risk of physician suicide. *Journal of the American Medical Association*.

[42] Preven DW (1981). Physician suicide. *Hillside Journal of Clinical Psychiatry*.

[43] Dumais A, Lesage AD, Alda M (2005). Risk factors for suicide completion in major depression: a case-control study of impulsive and aggressive behaviors in men. *American Journal of Psychiatry*.

[44] Bridge S (2006). Suicide prevention - targeting the patient at risk. *Australian family physician.*.

[45] Hawton K, Clements A, Simkin S, Malmberg A (2000). Doctors who kill themselves: a study of the methods used for suicide. *QJM - Monthly Journal of the Association of Physicians*.

[46] Cadman M, Bell J (1998). Doctors detected self-administering opioids in New South Wales, 1985–1994: characteristics and outcomes. *Medical Journal of Australia*.

